# Management of Head and Neck Paragangliomas: AGREE II Appraisal of Clinical Practice Guidelines

**DOI:** 10.1245/s10434-025-17682-2

**Published:** 2025-06-28

**Authors:** Deepak R. Lakshmipathy, Eric Winter, Christian Fritz, Om Balar, Aman Prasad, Alvaro Moreira, Karthik Rajasekaran

**Affiliations:** 1 Department of Otorhinolaryngology–Head & Neck Surgery, University of Pennsylvania, Philadelphia, PA USA; 2 Department of Medicine, University of Pennsylvania, Philadelphia, PA USA; 3 Department of Biology, The College of New Jersey, Ewing Township, NJ USA; 4 Department of Pediatrics, University of Texas Health Science Centre at San Antonio, San Antonio, TX USA; 5 Leonard Davis Institute of Health Economics, University of Pennsylvania, Philadelphia, PA USA

## Abstract

**Background:**

Clinical practice guidelines (CPGs) have recently been created to help standardize management of head and neck paragangliomas (HNPGLs) given their rarity and anatomic proximity to high-risk structures. The aim was to critically evaluate available CPGs using the Appraisal of Guidelines for Research and Evaluation (AGREE II) tool and to answer whether such guidelines are of sufficient quality.

**Methods:**

Electronic guideline databases were systematically searched until December of 2023. The inclusion criteria encompassed all CPGs that provided thorough HNPGL management recommendations. Non-English publications and prior versions of existing guidelines were excluded. Selected, relevant CPGs then were independently rated by four reviewers trained in AGREE II protocols over 23 key items and 6 overarching domains. Intraclass correlation coefficients also were calculated to assess interrater reliability.

**Results:**

Of 523 initially identified records, 7 CPGs met the inclusion criteria. Three CPGs were designated as high quality, with the remaining four considered as low quality. Generally, the CPGs did well in defining scope and purpose (84.33% ± 14.91%) and clearly presenting recommendations (77.98% ± 18.59%). However, the CPGs uniformly struggled in outlining stakeholder involvement (56.15% ± 16.25%), using evidence-based development (50.15% ± 23.64%), offering facile applicability (49.55% ± 17.58%), and delineating independence from outside influence (59.52% ± 39.71%). Interrater reliability was good to excellent across all domains.

**Conclusions:**

Most current CPGs on management of HNPGLs are of low quality and would significantly benefit from incorporating standardized evidence-gathering and recommendation-formation practices, systematic review experts, health economists, patient perspectives, and funding disclosures during future development.

Paragangliomas (also known as glomus tumors) are rare tumors derived from paraganglia adjacent to parasympathetic or sympathetic ganglia.^1,2^ Although they share a similar cellular origin, paragangliomas associated with the sympathetic system are predominantly localized to the adrenal gland (i.e., pheochromocytomas) or elsewhere along the sympathetic chain in the thorax (i.e., mediastinal paragangliomas) or abdomen (i.e., paragangliomas of the organ of Zuckerkandl).^2^ Parasympathetic paragangliomas, instead, mainly arise as head and neck paragangliomas (HNPGLs) in locations such as the carotid body, jugular bulb, and middle ear.^2^ Although HNPGLs are predominantly benign and non-functional (i.e., do not secrete hormones), their malignant variants can present some of the most difficult treatment conundrums.^3,4^ The variety of included subtypes combined with proximity to high-risk cranial nerves and vasculature can lead to adverse outcomes for poorly managed patients.^2,5,6^

Management options for HNPGL are diverse and rely on multidisciplinary teams including but not limited to otolaryngologists, geneticists, radiation oncologists, and vascular surgeons.^7^ The choice of observation, surgical resection, radiation, or chemotherapy is highly dependent one characteristics of the tumor and the patient.^6,8^ For example, mutations in SDHA, SDHD, and TMEM127 genes have been linked to hereditary variants of HNPGL that place afflicted patients at higher risk for the development of multiple tumors.^9^ This suggests that these patients may benefit from systemic treatments rather than targeted surgery or stereotactic radiosurgery.^10^ Furthermore, the less than 0.001% annual incidence of these tumors must be taken into consideration.^4,11^ Centers should have a sufficiently high volume of experience to reliably provide the care dictated by a HNPGL’s location, size, and type.^12–14^ Efforts have since been taken to share clinical pearls from experienced care teams across more medical centers.

Clinical practice guidelines (CPGs) have been growing rapidly in both number and focus for the past several decades.^15,16^ They aim to standardize care between different institutions by assembling a team of experts who systematically review and critically evaluate available evidence on a topic, publicly sharing their consensus recommendations.^16^

In theory, CPGs are a valuable resource for rare diseases such as HNPGL in which robust clinical trial data are not feasible, but their quality is rarely audited. This poses a significant problem for cases in which non-evidence-based CPGs influence direct patient care. A wide variety of guideline appraisal tools have been developed to combat this issue, with the updated version of the Appraisal of Guidelines for Research and Evaluation (AGREE II) tool the most widely adopted.^17,18^ The components the AGREE II instrument have been thoroughly tested and have demonstrated success in evaluating CPGs for a variety of head and neck tumors.^19–25^ Such appraisals have yet to be performed for CPGs related to HNPGLs.

We consequently sought to assess the quality of guidelines on the management of HNPGLs using the AGREE II instrument. By rigorously evaluating relevant guidelines, we hoped to objectively state whether current CPGs were developed in a trustworthy, evidence-based manner. We additionally aimed to stratify the different levels of available CPGs, highlight their strengths and weaknesses, and identify concrete areas of improvement for future guideline developers and methodologists.

## Methods

### Guideline Identification

All the authors agreed upon the protocol for this study before its initiation. A systematic literature search was performed across Embase, PubMed, Web of Science, and relevant professional society websites. The inclusion criteria encompassed all CPGs from database inception through December of 2023 that provided thorough HNPGL management recommendations. Non-English publications and prior versions of existing guidelines were excluded. Search terms consisted of the following combination of keywords, truncations, and Boolean operators: (((((head OR scalp) AND neck*) OR (extra* AND adrenal)) AND ((paraganglioma* OR (glomus* AND (tumor* OR neoplasm* OR cancer* OR adenoma* OR carcinoma*))))) OR "Paraganglioma, Extra-Adrenal"[Mesh] OR "Glomus Tumor"[Mesh]) AND ((clinical* AND practice AND guideline*) OR guideline* OR consensus OR recommendation* OR "Practice Guidelines as Topic"[Mesh]).

After the Preferred Reporting Items for Systematic Reviews and Meta-Analyses (PRISMA) protocols, duplicate records were first removed via Covidence systematic review software (Veritas Health Innovation, Melbourne, Australia).^26^ Two authors (D.L., E.W.) then independently voted on whether or not to include individual publications first by title and abstract and then by full-text screening. A third author (K.R.) independently resolved conflicting votes as needed.

### Guideline Evaluation

Relevant general characteristics of selected guidelines were extracted before appraisal. Four reviewers (D.L., E.W., C.F., O.B.) trained in AGREE II protocols independently rated each selected CPG based on 23 key items and 6 overarching domains (Table 1). Each evaluator assigned a score from 1 (strongly disagree) to 7 (strongly agree) for each item based on objective criteria provided in the AGREE II manual (publicly available at <https://www.agreetrust.org>). These ratings were used to calculate scaled domain scores via the following formula:

**Table 1:** Components of the AGREE II instrument

Domain 1: Scope and purpose	
1.	The overall objective(s) of the guideline is (are) specifically described.
2.	The health question(s) covered by the guideline is (are) specifically described.
3.	The population (i.e., patients, public) to whom the guideline is meant to apply is specifically described.

Domain 2: Stakeholder involvement	
4.	The guideline development group includes individuals from all relevant professional groups.
5.	The views and preferences of the target population (i.e., patients, public) have been sought.
6.	The target users of the guideline are clearly defined.

Domain 3: Rigor of development	
7.	Systematic methods were used to search for evidence.
8.	The criteria for selecting the evidence are clearly described.
9.	The strengths and limitations of the body of evidence are clearly described.
10.	The methods for formulating the recommendations are clearly described.
11.	The health benefits, side effects, and risks have been considered in formulating the recommendations.
12.	There is an explicit link between the recommendations and the supporting evidence.
13.	The guideline has been externally reviewed by experts before its publication.
14.	A procedure for updating the guideline is provided.

Domain 4: Clarity of presentation	
15.	The recommendations are specific and unambiguous.
16.	The different options for management of the condition or health issue are clearly presented.
17.	Key recommendations are easily identifiable.

Domain 5: Applicability	
18.	The guideline describes facilitators and barriers to its application.
19.	The guideline provides advice and/or tools on how the recommendations can be put into practice.
20.	The potential resource implications of applying the recommendations have been considered.
21.	The guideline presents monitoring and/or auditing criteria.

Domain 6: Editorial independence	
22.	The views of the funding body have not influenced the content of the guideline.
23.	Competing interests of guideline development group members have been recorded and addressed.

AGREE II, Appraisal of Guidelines for Research and Evaluation

$$\text{Scaled domain score}= \frac{\text{obtained score}-\text{minimum possible score}}{\text{maximum possible score}-\text{minimum possible score}} \times 100\text{\%}$$

The obtained score refers to the sum of all reviewer scores for all items contained within a domain. The minimum possible score and maximum possible score refer to the same sum but assume all reviewer scores are 1 (the minimum rating) or 7 (the maximum possible rating), respectively.

Scaled domain scores were used to confer overall quality appraisals. A 60% scaled domain score threshold was used based on prior literature.^27–30^ Specifically, CPGs with ≥5 domains, 3 or 4 domains, and ≤2 domains with scaled domain scores of 60% or higher were deemed as high, moderate, and low quality, respectively. Descriptive statistics (i.e., means and standard deviations) for individual CPGs and domains also were calculated using Microsoft Excel (version 16.81, Microsoft Corporation, Redmond, WA, USA).

### Interrater Reliability Assessment

Intraclass correlation coefficients (ICCs) were used to objectively quantify interrater reliability for each domain. An averaged two-way random-effects model was chosen given its aptitude for accurately representing multiple, similarly-trained human evaluators.^31^ The ICCs were calculated via R (version 4.3.1; R Core Team, Vienna, Austria) using the “psych” R package (version 2.4.1; Revelle, Evanston, IL, USA) in RStudio (version 2023.06.1+524l, Posit Software, Boston, MA, USA). Per the literature, ICCs higher than 0.90, between 0.90 and 0.75, between 0.75 and 0.50, and lower than 0.50 were designated as excellent, good, moderate, and poor, respectively.^32^ Descriptive statistics (i.e., 95% confidence intervals) also were calculated for each domain’s ICC using the same software.

## Results

### Selected CPGs

**Fig. 1:**
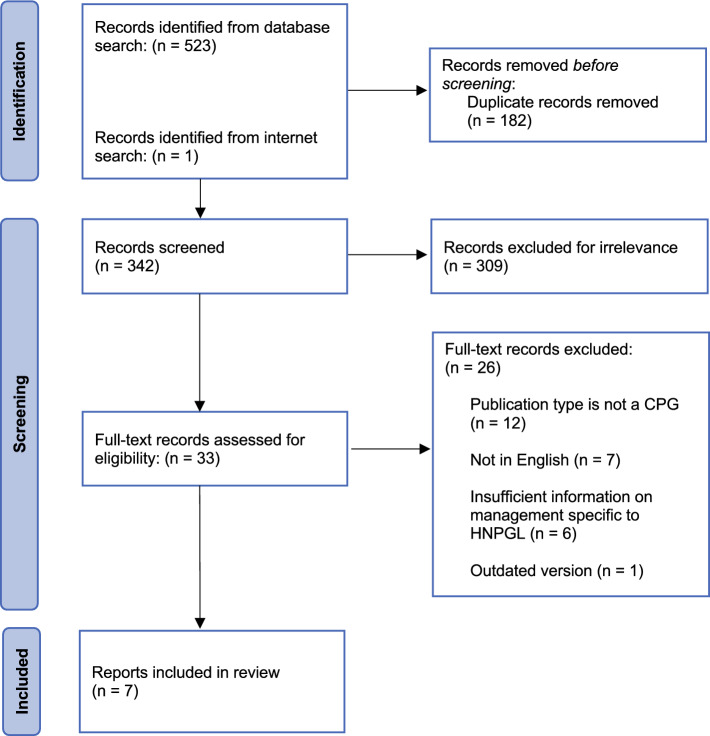
Systematic search algorithm used to identify relevant CPGs following Preferred Reporting Items for Systematic Reviews and Meta-Analyses (PRISMA) protocols. CPGs, clinical practice guidelines; HNPGL head and neck paraganglioma

As shown in Fig. 1, 524 records were retrieved in the initial searching. These were narrowed to seven CPGs after the screening protocol described earlier.^33–39^ The properties of each guideline are outlined in Table 2. All the guidelines were published within the last 4 years excluding the guidelines from a Croatian British multidisciplinary collaboration (CBMC) published in 2009.^34^ The majority of the guidelines (86%) arose from European countries, but North America, Asia, and Australia also were represented.^33–39^

**Table 2:** Properties of selected clinical practice guidelines

First author, year	Development group(s)	Abbreviation	Region(s) of origin	Intended users	Evidence base	Guideline focus
Lloyd, 2020^33^	British Skull Base Society	BSBS	United Kingdom	Multidisciplinary	Expert consensus, systematic literature review	Diagnosis and management of HNPGL
Gjuric, 2009^34^	Croatian British Multidisciplinary Collaboration	CBMC	Croatia, United Kingdom	Multidisciplinary	Expert consensus, literature review	Diagnosis and management of HNPGL
Lenders, 2020^35^	European Society of Hypertension	ESH	Europe	Multidisciplinary	Expert consensus, literature review	Diagnosis, management, and future directions of research for pheochromocytoma and paraganglioma
Taïeb, 2023^36^	Multidisciplinary Global Medical Institute Collaboration	MGMIC	North America, Europe, Australia, Asia	Multidisciplinary	Expert consensus, systematic literature review	Diagnosis and management for pheochromocytoma and paraganglioma in patients with pathologic SDHD mutations
Garcia-Carbonero, 2021^37^	Multidisciplinary Spanish Medical Society Collaboration	MSMSC	Spain	Multidisciplinary	Expert consensus, literature review	Diagnosis and management for pheochromocytoma and paraganglioma
Fishbein, 2021^38^	North American Neuroendocrine Tumor Society	NANETS	North America	Multidisciplinary	Expert consensus, systematic literature review	Diagnosis, management, and future directions of research for metastatic and/or unresectable pheochromocytoma and paraganglioma
Dissaux, 2021^39^	French Society for Radiation Oncology	SFRO	France	Multidisciplinary	Expert consensus, literature review	Indications and treatment of benign intracranial tumors with radiosurgery and radiotherapy

HNPGL, head and neck paraganglioma; SDHD, succinate dehydrogenase subunit D

All the CPGs were intended for a multidisciplinary user base and used a combination of expert consensus and literature review to generate their recommendations. Although all the guidelines provided thorough recommendations on the management of HNPGL, their specific focuses were more heterogeneous. Some were solely designed for HNPGL whereas other CPGs provided discourse on similar tumors such as pheochromocytomas.^33–39^

### AGREE II Appraisals

Scaled domain scores for each of the seven guidelines are shown in Table 3. The guideline arising from a multidisciplinary global medical institute collaboration (MGMIC) had the highest overall mean scaled domain score (85.45%), and the CBMC guideline had the lowest (38.92%).^34,36^ Regarding specific domains, the highest scaled domain score of 100% was achieved by the British Skull Base Society (BSBS) and MGMIC guidelines for the editorial independence and clarity of presentation domains, respectively.^33,36^ The lowest scaled domain score of 0.00% was given to the CBMC guideline for the editorial independence domain.^34^

**Table 3:** Scaled domain scores and quality appraisals stratified by guideline

	Domain 1:	Domain 2:	Domain 3:	Domain 4:	Domain 5:	Domain 6:		
Guideline	Scope and purpose (%)	Stakeholder involvement (%)	Rigor of development (%)	Clarity of presentatio*n* (%)	Applicability (%)	Editorial independence (%)	Mean overall score (%)	Quality appraisal
BSBS^33^	97.22	70.83	72.92	76.39	73.96	100.00	81.89	High
CBMC^34^	68.06	36.11	29.69	56.94	42.71	0.00	38.92	Low
ESH^35^	91.67	44.44	31.25	88.89	39.58	50.00	57.64	Low
MGMIC^36^	94.44	66.67	84.90	100.00	72.92	93.75	85.45	High
MSMSC^37^	90.28	59.72	43.75	52.78	41.67	93.75	63.66	Low
NANETS^38^	90.28	76.39	63.54	97.22	48.96	62.50	73.15	High
SFRO^39^	58.33	38.89	25.00	73.61	27.08	16.67	39.93	Low
Mean ± SD	84.33 ± 14.91	56.15 ± 16.25	50.15 ± 23.64	77.98 ± 18.59	49.55 ± 17.58	59.52 ± 39.71	84.33 ± 14.91	

BSBS, British Skull Base Society; CBMC, Croatian British Multidisciplinary Collaboration; ESH, European Society of Hypertension; MGMIC, Multidisciplinary Global Medical Institute Collaboration; MSMSC, Multidisciplinary Spanish Medical Society Collaboration; NANETS, North American Neuroendocrine Tumor Society; SFRO, French Society for Radiation Oncology; SD, standard deviation

All the CPGs performed generally well in the scope and purpose (84.33% ± 14.91%) and clarity (77.98% ± 18.59%) of presentation domains. However, they uniformly struggled in outlining stakeholder involvement (56.15% ± 16.25%), using evidence-based development (50.15% ± 23.64%), offering facile applicability (49.55% ± 17.58%), and delineating independence from outside influence (59.52% ± 39.71%). The BSBS, MGMIC, and North American Neuroendocrine Tumor Society (NANETS) guidelines were designated as high quality, whereas the guidelines from CBMC, European Society of Hypertension (ESH), multidisciplinary Spanish Medical Society collaboration (MSMSC), and French Society for Radiation Oncology (SFRO) were deemed as low quality. No guidelines received a moderate quality appraisal.

### Interrater Reliability

The ICCs calculated across the four evaluators (D.L., E.W., C.F., O.B.) and six domains are given in Table 4. The interrater reliability was excellent across the majority (67%) of the domains and good for the clarity of the presentation and applicability domains. No moderate or poor interrater reliabilities were observed. The 95% confidence intervals were narrow for stakeholder involvement, rigor of development, and editorial independence. No CIs included the null hypothesis value of 0.00.

**Table 4:** Intraclass correlation coefficients and associated interrater reliabilities stratified by domain

AGREE II domain	ICC (95% CI)	Interrater reliability
Domain 1: Scope and purpose	0.91 (0.47–1.00)	Excellent
Domain 2: Stakeholder involvement	0.99 (0.97–1.00)	Excellent
Domain 3: Rigor of development	0.99 (0.98–1.00)	Excellent
Domain 4: Clarity of presentation	0.86 (0.32–1.00)	Good
Domain 5: Applicability	0.82 (0.26–0.99)	Good
Domain 6: Editorial independence	0.98 (0.78–1.00)	Excellent

AGREE II, Appraisal of Guidelines for Research and Evaluation; ICC, intraclass correlation coefficient; CI, confidence interval

## Discussion

To our knowledge we present the first quality appraisal of CPGs on the management of HNPGLs. We accomplished this using the AGREE II instrument, highlighting that most current guidelines are of low quality. This is a concerning finding given the nuances of HNPGL management and the risks of non-evidence-based guidance for patient outcomes. Although the MGMIC, BSBS, and NANETS guidelines are of high quality and can serve as valuable developmental frameworks, additional action items should be implemented to improve future guidelines on the management of HNPGL.

### Reliability of the Study Protocol

It is important to first analyze whether the findings of this study are based on trustworthy research methods. Beginning with the literature search, PRISMA protocols were purposely used to adhere to a globally recognized standard, provide transparency about methodology, and allow for reproducibility of results.^26^ Inclusion of multiple electronic databases, internet searching, and broad search terms provided safeguards against missing relevant CPGs. Independent screening and voting by multiple authors helped to limit personal selection bias.

The five different components and diverse focuses among the guidelines displayed in Table 1 also suggest that the included CPGs are representative.^33–39^ Furthermore, their recommendations likely reflect current evidence given that almost all were released in the past 5 years and included mentions of new gene-based management algorithms. The AGREE II evaluations appear to be similarly valid. The ideal number of reviewers recommended by the AGREE II manual (i.e., 4) was used together with good to excellent interrater reliabilities across every domain.

### Current Guideline Strengths

The current CPGs performed uniformly well in the scope, purpose, and clarity of the presentation domains. These strengths are particularly valuable in the context of HNPGL. Previously we highlighted how HNPGL encompasses multiple tumor types in complex anatomic locations.^2^ Clearly delineating scope and purpose is therefore critical because it can help target users to understand both the depth and limitations of each guideline. For instance, radiation oncologists seeking guidance on radiation doses for carotid body tumors can quickly see if this topic is included within a CPG and whether its recommendations can be applied to patients with malignant variants. This aptitude manifests similarly for clarity of presentation. Straightforward displays of recommended management algorithms and alternatives allow members of multidisciplinary HNPGL care teams to understand one another’s decision-making more easily. Preventing delays in treatment can subsequently improve outcomes for patients with fast-growing neoplasms. With this in mind, guideline authors would do well to continue excelling in both practices.

### Current Guideline Weaknesses

Weaknesses in rigor of development and applicability were apparent among the current guidelines. Deficiencies in the former category are especially concerning given its connection with evidence-based medicine. The rigor of the development domain specifically assesses whether CPGs systematically gathered evidence, critiqued collected data, transparently used objective criteria in forming expert consensus, incorporated peer review, and outlined protocols for updating recommendations. Understandably, lacking any of these components can compromise the quality of a CPG’s recommendation. This can be especially dangerous for rare pathologies such as HNPGL, for which clinical experience is limited. Shortcomings in applicability can be equally damaging. The applicability domain tests whether CPG recommendations can be carried out realistically and how impact may be measured. Again, for uncommon tumors such as HNPGL, access to centers capable of carrying out treatments must be considered.^12–14^ Whether it be for specific genetic testing or operations by experienced surgeons, CPGs should provide guidance on when limited resources necessitate referring patients elsewhere. It is paramount that future guideline authors spend significant time improving every aspect of both domains.

The deficiencies in stakeholder involvement and editorial independence share a different pattern. Upon closer inspection of individual reviewer ratings, the average of both domains was brought down by low scores for singular items.

Regarding stakeholder involvement, many guidelines did not incorporate perspectives from their target population during recommendation formation. It is unclear why this may have occurred. Possible reasons include the already large time costs associated with forming CPGs coupled with the intensive resources needed to properly sample patient opinion.^40–43^ Nevertheless, public opinion should be incorporated to better define topics of interest and provide valuable information on quality of life following specific treatment methods.^44,45^

For editorial independence, multiple CPGs lacked statements about how funding bodies did not influence guideline contents. This represents an obvious flaw. Target users should have confidence that CPG recommendations are free from undue influence and based on evidence alone. Addressing both specific shortcomings in future iterations would therefore improve overall CPG quality.

### Possible Directions for Improvement

Several concrete recommendations for improvement can be synthesized from the aforementioned strengths and weaknesses. First, the high-quality MGMIC, BSBS, and NANETS guidelines should serve as developmental frameworks for guideline authors and methodologists alike.^33,36,38^ Second, guideline creators should consider including methodology experts such as library scientists, systematically reviewing evidence per PRISMA protocols and forming recommendations via globally accepted standards (i.e., the Delphi technique) to improve rigor of development.^26,46^ Third, applicability could be strengthened by adding health economists to development boards for assistance in complex cost analysis and implementation strategy.^47,48^ Fourth, gathering and incorporating perspectives from HNPGL patients could shore up weaknesses in stakeholder involvement. Fifth, clear statements regarding independence of guideline recommendations from funding sources would ensure a high standard of editorial independence.

## Study Limitations

Several limitations of this study should be acknowledged. Beginning with guideline identification, a degree of selection bias was introduced by the exclusion of seven non-English articles. This likely was mitigated by close adherence to PRISMA protocols, but such bias cannot be eliminated altogether. Likewise, the AGREE II instrument is an inherently subjective tool. Although all domains had good to excellent interrater reliabilities, personal biases may have partially influenced reviewer ratings. A similar trend existed among the scaled domain scores. By looking closely at the formula listed in the Methods section, the lack of accountability regarding domain size becomes apparent. Domains such as “rigor of development” with eight items are subsequently less susceptible to wide variations in scoring versus domains such as editorial independence, with two items. This may indicate that scaled domain scores for larger domains are more accurate than smaller ones. Finally, the AGREE II device is designed to assess only the methodologic quality of a guideline.^18^ Guidelines deemed as low quality still may provide valuable pearls of clinical judgment, especially on topics lacking higher-level evidence.^49^

## Conclusions

This report provides the first known quality appraisal of CPGs on the management of HNPGLs via the AGREE II instrument. We highlight how most current CPGs are of low quality and that the high-quality MGMIC, BSBS, and NANETs guidelines should serve as developmental frameworks.^33,36,38^ The current guidelines do well in outlining their scope and purpose and in clearly presenting their recommendations, but struggle in domains of stakeholder involvement, rigor of development, applicability, and editorial independence. These CPGs could significantly improve by gathering evidence and forming recommendations in a standardized manner, adding systematic review experts and health economists as authors, incorporating patient perspectives during development, and adequately acknowledging funding sources. Such changes could raise not only guideline trustworthiness but also global standards of care for HNPGL patients.
